# The retrospective study of the metabolic patterns of BCG-vaccination in type-2 diabetic individuals in COVID-19 infection

**DOI:** 10.3389/fimmu.2023.1146443

**Published:** 2023-04-05

**Authors:** Najeha R. Anwardeen, Farhan S. Cyprian, Hadi M. Yassine, Asmaa A. Al-Thani, Abdallah M. Abdallah, Mohamed M. Emara, Mohamed A. Elrayess

**Affiliations:** ^1^ Biomedical Research Center (BRC), QU Health, Qatar University, Doha, Qatar; ^2^ Department of Basic Medical Sciences, College of Medicine, QU Health, Qatar University, Doha, Qatar; ^3^ College of Health Sciences, QU Health, Qatar University, Doha, Qatar

**Keywords:** COVID - 19, SARS – CoV – 2, diabete mellitus, BCG vaccination, sarcosine, metabolomics, arachidonic acid (AA)

## Abstract

**Background:**

The cross-protective nature of Bacillus Calmette-Guerin (BCG) vaccine against SARS-CoV-2 virus was previously suggested, however its effect in COVID-19 patients with type 2 diabetes (T2D) and the underlying metabolic pathways has not been addressed. This study aims to investigate the difference in the metabolomic patterns of type 2 diabetic patients with BCG vaccination showing different severity levels of COVID-19 infection.

**Methods:**

Sixty-seven COVID-19 patients were categorized into diabetic and non-diabetic individuals who had been previously vaccinated or not with BCG vaccination. Targeted metabolomics were performed from serum samples from all patients using tandem mass spectrometry. Statistical analysis included multivariate and univariate models.

**Results:**

Data suggested that while BCG vaccination may provide protection for individuals who do not have diabetes, it appears to be linked to more severe COVID-19 symptoms in T2D patients (p = 0.02). Comparing the metabolic signature of BCG vaccinated T2D individuals to non-vaccinated counterparts revealed that amino acid (sarcosine), cholesterol esters (CE 20:0, 20:1, 22:2), carboxylic acid (Aconitic acid) were enriched in BCG vaccinated T2D patients, whereas spermidine, glycosylceramides (Hex3Cer(d18:1_22:0), Hex2Cer(d18:1/22:0), HexCer(d18:1/26:1), Hex2Cer(d18:1/24:0), HexCer(d18:1/22:0) were higher in BCG vaccinated non- T2D patients. Furthermore, data indicated a decrease in sarcosine synthesis from glycine and choline and increase in spermidine synthesis in the BCG vaccinated cohort in T2D and non-T2D groups, respectively.

**Conclusion:**

This pilot study suggests increased severity of COVID-19 in BCG vaccinated T2D patients, which was marked by decreased sarcosine synthesis, perhaps *via* lower sarcosine-mediated removal of viral antigens.

## 1 Introduction

Severe acute respiratory syndrome coronavirus 2 (SARS-CoV-2) emerged as a novel human pathogen that led to world’s leading pandemic in the year 2019 (1). Bacille Calmette-Guerin (BCG) vaccination at infancy was suggested to alleviate the severe impact of the pandemic in some countries (2).

BCG vaccine was developed by Albert Calmette and Camile Guérin to protect against *Mycobacterium tuberculosis* infection, otherwise called Tuberculosis (TB). By procuring the attenuated strain of the bacteria *Mycobacterium bovis*, followed by several clinical trials, BCG vaccination has proved to be dependable (3). Much later into its introduction, it was reported that BCG was not only effective against *Mycobacterium tuberculosis* but also protective against several other infections in various studies (4, 5). BCG administration has shown a 45% reduction in mortality rate in West Africa (6) and decreased child mortality in Sweden (7) possibly due to its non-specific protective nature. These pilot studies suggested that BCG vaccine was responsible for lower incidence of neonatal sepsis and pulmonary infections that decreased infant mortality (8). Other examples include protection against yellow fever virus (9), HPV (human papilloma virus) (10), HSV (herpes simplex virus) (11), RSV (respiratory syncytial virus) (12) and Influenza A (H1N1) (13, 14). Automatously, the cross-protective effect of BCG vaccination can be delineated, partially, by training innate immunity based on temporary epigenetic reprogramming of macrophages, which enables them to produce more inflammatory cytokines, triggering powerful immunological responses (15). This innate immunity training mechanism was explained several years ago (9). Furthermore, the BCG vaccine also offers protection against immunological disorders including type 1 diabetes and multiple sclerosis (16–18). The mechanics underlying these numerous advantages are still being actively researched by scientists.

The controversy over whether the BCG vaccine can impact the immune response against the virus or other unrelated diseases was reignited in 2019 with the onset of the severe acute respiratory syndrome coronavirus (SARS-CoV-2) pandemic (15). The BCG vaccine is the only vaccine licensed against TB and due to the above observations regarding its cross-protectivity, it is proposed that many low- and lower-middle-income nations would probably have the infrastructure and medical staff necessary to distribute the novel BCG-based COVID-19 vaccine on a large-scale. This shows that BCG may be able to get over any remaining obstacles to vaccine adoption in nations with weak health-care systems and potentially catastrophic coronavirus consequences (14, 19). Although the effect of BCG against COVID-19 infection remains controversial due to minimal number of published clinical trials (2).

Currently, the impact of BCG vaccinations seems to be largely hypothetical or non-specific in decreasing the severity of COVID-19 infection. Our previous study suggested a difference in the metabolic signature of Type 2 diabetic patients with COVID-19 infection compared to non-diabetic population (20). In general, diabetic patients are at a greater risk of disease progression when infected with SARS-CoV-2. Compared to SARS-CoV-2-infected non-diabetic people, diabetics have a higher rate of hospital admission, severe pneumonia, and worse mortality rate (21, 22). A meta-analysis revealed that diabetes raises the probability of severity by 2.3 times and the risk of the COVID-19-related death by 2.5 times, making it a main contributing factor to the poor prognosis in COVID-19 (23). In this context, the effect of BCG vaccination should be evaluated in diabetic patients with COVID-19. Since an altered metabolic profile is evident in COVID-19 patients, there might be altered expression of disease in diabetic patients with respect to BCG vaccination. The objective of this study is to investigate the difference in the metabolic patterns of type 2 diabetic patients with BCG vaccination showing different levels of COVID-19 infection and identify the underlying metabolic pathways.

## 2 Methods

### 2.1 Study design

**Figure 1:**
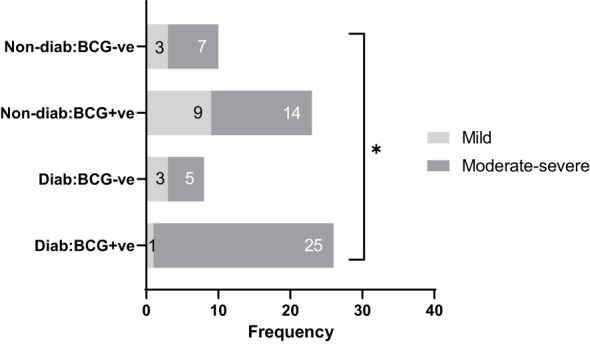
Study participants classified by disease severity (mild and moderate-severe), diabetes status (diabetic, non-diabetic), and BCG vaccination status (positive, negative). * signifies p-value <0.05.

The study included 67 patients diagnosed with SARS-CoV-2 infection at Hamad Medical Corporation (HMC, Doha) between the period of June 2020 and March 2021. Participants were included if they were between 35 and 65 years of age, had a positive SARS-CoV-2 PCR test result (with a CT value <30), and were residents of Qatar. Upper respiratory tract specimens, specifically throat and nasopharyngeal swabs, were collected and tested for SARS-CoV-2 using the TaqPath COVID-19 Combo Kit (Thermo Fisher Scientific, Waltham, Massachusetts) or Cobas SARS-CoV-2 Test (Roche Diagnostics, Rotkreuz, Switzerland). The study recruited all consenting patients from Hammad Medical Corporation. Protocols were approved by the Institutional Review Boards (IRBs) of HMC (MRC-01-20-145) and Qatar University (QU-IRB 1289-EA/20). The patients were clinically identified as symptomatic, mild, moderate and severe based on WHO’s COVID-19 guidelines (24). For analysis, the cohort were categorized into Type 2 diabetic (T2D) and non-diabetic (non-T2D) and on the basis of BCG vaccination status as: BCG+/T2D (n=26), BCG-/T2D (n=8), BCG+/non-T2D (n=23), BCG-/non-T2D (n=10) and further dichotomized as mild and moderate-severe cases. The moderate and severe cases were grouped to differentiate RT PCR positive cases with no clinical findings (mild) from those with respiratory symptoms, pulmonary involvement (moderate), and SpO2 <94% on room air (severe). **Figure 1** shows the categorization of study participants in each of the groups. Blood samples were drawn during the diagnosis, before quarantine, or hospitalization. Patients exhibiting moderate to severe disease symptoms were given inpatient treatment. Two patients in the severe group died due to respiratory failure. Data regarding the clinical parameters were retrieved from the hospital’s healthcare system following patients consent, which includes Body Mass Index (BMI), SARS-CoV-2 viral load, complete blood count (CBC), and specific blood tests.

### 2.2 Metabolomics

Using the Biocrates MxP^®^ Quant 500 Kit (Biocrates, Innsbruck, Austria), assessed by tandem mass spectrometry at the Fraunhofer Institute for Toxicology and Experimental Medicine, targeted metabolomics was performed on serum samples obtained from all subjects between 24 and 48 h after diagnosis. As part of the MetIDQ™ MetaboINDICATOR™ module created especially for MxP^®^ Quant 500 kit data, 630 metabolites were evaluated. Additionally, 202 metabolite-indicators were created from combinations of metabolite measurements that encapsulate significant biological processes, for example indicators of enzymatic activity (substrate/product metabolite ratio) and sum of functionally or structurally similar metabolites. Quantification of lipids and small molecules were performed using Flow injection Analysis Tandem Mass Spectrometry (FIA-MS/MS) and liquid chromatography-tandem mass spectrometry (LC-MS/MS) respectively, using 5500 QTRAP^®^ instrument triple quadrupole mass spectrometer (AB Sciex, Darmstadt, Germany). The “_” signifies that the fatty acid residue locations (sn-1/sn-2/sn-3) for the listed triacylglycerols are unknown. Potential isomers are described by the manufacturer: https://biocrates.com/wp-content/uploads/2020/02/Biocrates_Q500_isomers_isobars.pdf.

### 2.3 Statistical analysis

The metabolite measurements were log transformed and orthogonal partial least squares discriminant analysis (OPLS-DA) was performed using SIMCA (v.16). Linear regression model was run separately for T2D and non-T2D groups using RStudio version 4.2.1 with metabolites depicted as y-variable against BCG vaccination status while correcting for age and gender to deal with the imbalanced data. The nominal p-values were corrected for multiple testing using the false discovery rate (FDR) method. Metabolite classes were defined by Biocrates and those with more than three numbers were selected for functional enrichment analysis, which was conducted on list of metabolites ordered by p-value from the linear regression models using one-way Wilcoxon sum of ranks test and adjusted for multiple testing using FDR method. Metabolism indicators were excluded in functional enrichment analysis.

## 3 Results

### 3.1 Clinical characteristics of participants

As depicted in **Table 1**, COVID-19 patients with different severity levels (categorized as mild, moderate-severe) were classified according to their previous BCG vaccination and diabetes mellitus statuses. There was a significant difference between the 4 groups in terms of COVID-19 severity (p <0.05). Out of 34 diabetic patients, individuals with BCG vaccination (25, 96.15%) had higher moderate-severe cases than non-BCG group (5, 62.5%) (p < 0.05). On the other hand, in the non-diabetic population (n = 33) there was no significant difference between BCG vaccinated groups (14, 61%) and its non-vaccinated counterpart (7, 70%) with regard to COVID-19 severity. The comorbidities associated with diabetes and COVID-19 (hypertension, COPD, cardiovascular disease, cerebrovascular disease, and asthma) are depicted in **Table 1** which shows no significant difference in their proportions among the study populations. A higher incidence of dyslipidemia was observed among diabetic individuals compared to non-diabetic individuals (p 0.0046). However, further investigation revealed no significant difference in the prevalence of dyslipidemia between BCG-positive and BCG-negative diabetic individuals (p 0.409).

**Table 1:** Demographic characteristics of study participants categorized by diabetic status and BCG vaccination (positive and negative).

	Diabetic		Non-diabetic		
	BCG positive (25)	BCG negative (8)	BCG positive (23)	BCG negative (10)	p
COVID-19 severity					
Mild	1 (4%)	3 (37.5%)	9 (40%)	3 (30%)	**0.02**
Moderate-Severe	25 (96%)	5 (62.5%)	14 (60%)	7 (70%)	
Age	53.5 (48.5-2.5)	53 (50.5-56.5)	44 (37-49.5)	59 (47.75-62.5)	**0.004**
Gender					
Female: Male	5:21	0:8	0:23	0:10	**0.036**
BMI	30.45 (5.13)	29.58 (4.20)	28.32 (4.35)	28.94 (5.27)	0.681
Hypertension					
Yes: No	17:9	2:6	8:15	4:6	0.061
Cardiovascular disease					
Yes: No	1:25	0:8	0:23	0:10	0.659
Asthma					
Yes: No	3:23	0:8	2:21	0:10	0.546
COPD					
Yes: No	2:24	0:8	0:23	0:10	0.354
Dyslipidemia					
Yes: No	8:18	4:4	0:23	1:9	0.005
Cerebrovascular disease					
Yes: No	0:26	0:8	0:23	0:10	NA
White blood cell count (WBC) [x10^3/uL]	6.6 (5.275-10.225)	6.5 (5.075-7.2)	6 (4.425-7.975)	6.1 (4.925-6.85)	0.475
Red blood cell count (RBC) [x10^6/uL]	5 (4.525-5.575)	5.1 (4.925-5.3)	5.15 (4.9-5.4)	4.9 (4.55-5.075)	0.549
Hemoglobin (Hgb) [g/dL]	12.75 (11.95-14.65)	14.1 (13.5-14.625)	14.75 (13.425-15.425)	14.15 (12.75-14.85)	0.138
Absolute neutrophil count (ANC) [x10^3/uL]	4.95 (3.0425-8.2)	3.65 (2.825-5.875)	2.7 (2.10-5.2)	3.55 (2.875-4.45)	0.181
Lymphocyte count [x10^3/uL]	1.48 (0.63)	1.45 (0.56)	1.85 (0.83)	1.53 (0.86)	0.315
Platelet [10^9/L]	265 (218-342)	264 (200.75-323.75)	247 (213.25-300.75)	252 (178-326.75)	0.844
Uric acid [umol/L]	299 (224-349)	246 (197-322)	353.5 (293.5-391.25)	300.5 (298.25-302.75)	0.268
Urea [mmol/L]	5.7 (4.2-7.775)	5.7 (4.575-9.225)	4.75 (3.65-6.1925)	3.8 (2.95-6.2)	0.296
Creatinine [umol/L]	78 (63-95.25)	76.5 (74-95.25)	87.5 (80-92.5)	77.5 (66.75-85.25)	0.478
Bilirubin [mg/dL]	8.4 (5-13)	6 (5-10.75)	8.5 (6-12.75)	9 (6.5-10.075)	0.823
Alkaline phosphatase (ALP) [U/L]	81.9 (71-125)	89 (61-117)	75.5 (62.25-94.25)	93 (72-97)	0.483
Alanine aminotransferase (ALT) [U/L]	25 (20-59)	31 (25-53)	35 (19.75-47)	25 (19-35.5)	0.61
Aspartate aminotransferase (AST) [U/L]	24.5 (18.5-47.25)	24 (22-52)	25 (21-32)	27 (21.5-29)	0.996
Lactate dehydrogenase (LDH) [U/L]	370 (142)	329.6 (120.1)	398.6 (156.3)	237.2 (58.65)	0.229
Glucose	8.3 (6.4-10.575)	9.2 (7-9.95)	5.5 (4.9-5.8)	7.15 (6.05-8.675)	**<0.001**
Lactic acid [mmol/L]	1.4 (1.075-1.65)	1.55 (1.475-1.625)	1.35 (1.075-1.475)	1.9 (1.55-2)	0.643
COVID-19 Average Cycle Threshold (CT)	24.65 (5.76)	25.89 (6.71)	24.45 (6.85)	26.43 (4.76)	0.837

Bold characters signify p<0.05. NA, not applicable.

### 3.2 Metabolic signature of BCG vaccination in diabetic and non-diabetic groups with COVID-19 infection

**Figure 2:**
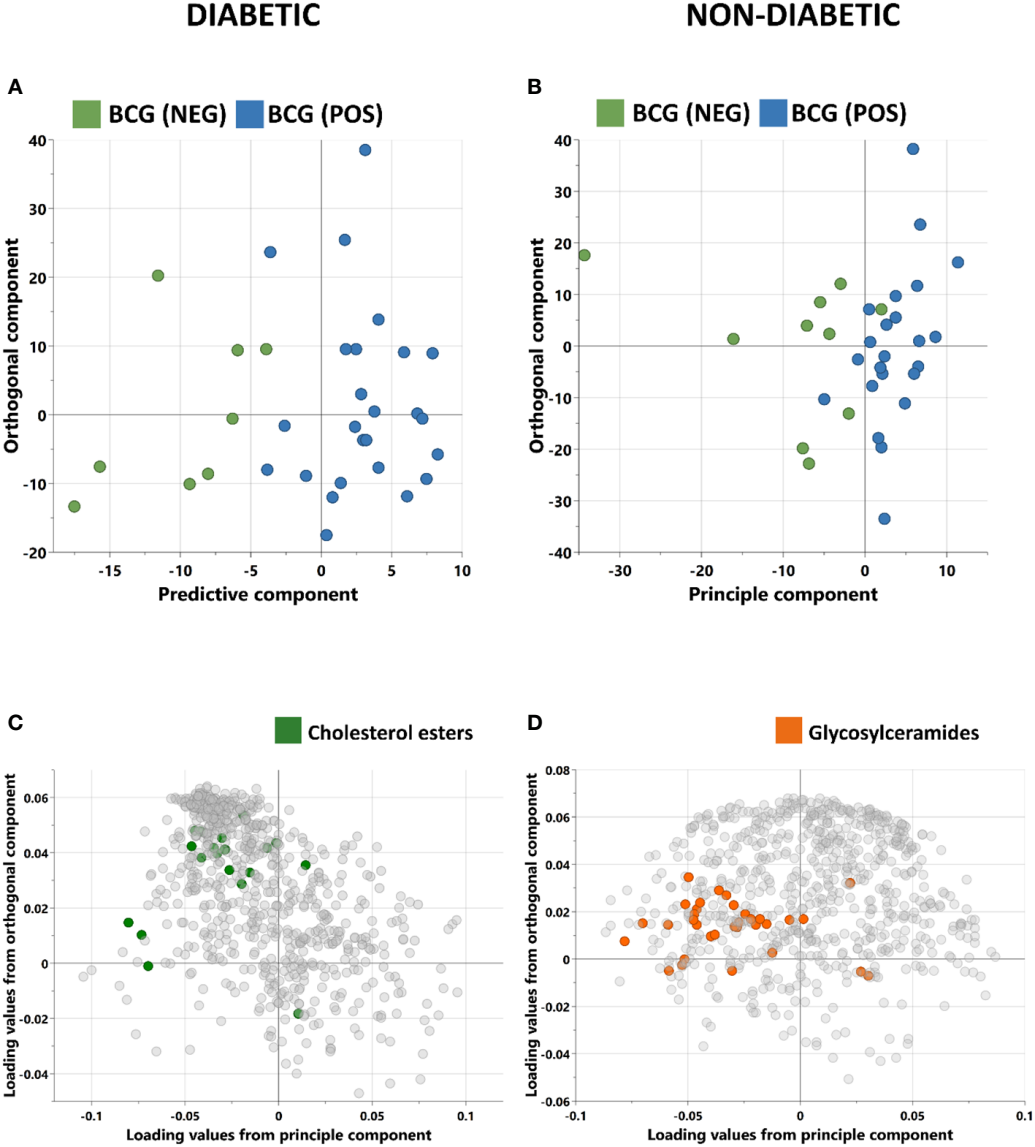
Score plots of OPLS-DA models discriminating between BCG vaccinated (BCG-pos) and non-vaccinated (BCG-neg) patients with COVID-19 infection. The analysis was performed separately for **(A)** Diabetic individuals **(B)** non-diabetic individuals. OPLS-DA identified one predictive and one orthogonal component for each model. **(C, D)** are the respective loading plots for diabetic and non-diabetic COVID-19 patients with highlighted enriched pathways.

Orthogonal partial least squares discriminant analysis (OPLS-DA) was conducted to capture the metabolic profile of BCG positive vs BCG negative with different levels of COVID-19 severity in diabetic and non-diabetic individuals. Data revealed a clear separation between BCG positive and BCG negative in diabetic patients (R2Y = 0.69) (**Figure 2A**), and in non-diabetic patients (R2Y = 0.45) (**Figure 2B**). The corresponding loadings plot for each OPLS-DA models revealed lower abundance of cholesterols esters/glycosylceramides in BCG-positive diabetic (**Figure 2C**)/non-diabetic (**Figure 2D**) patients, respectively.

### 3.3 Metabolites associated with BCG vaccination in T2D patients

**Figure 3:**
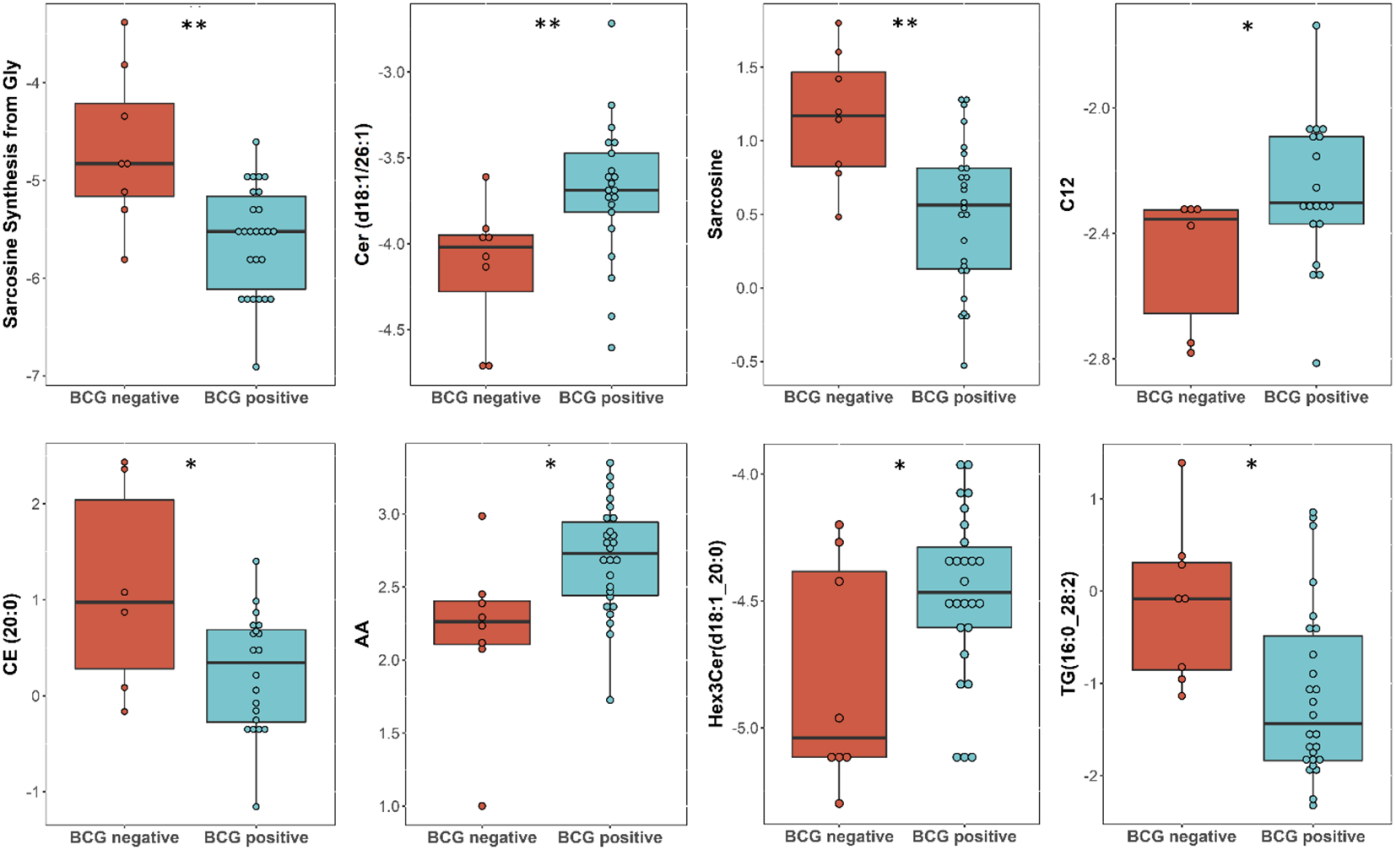
Metabolites associated with BCG vaccination in T2D patients with different levels of COVID-19 severity. **/* signifies <0.01/<0.05 p value.

Targeted metabolomics of serum samples from 34 diabetic patients was employed to elucidate the metabolites that differentiated between BCG-positive and BCG-negative individuals with COVID-19. Linear regression analysis provided several nominally significant (p < 0.01) metabolic changes between the two groups in diabetic individuals (**Table 2**). The altered metabolic profile involved decreased level of sarcosine, metabolic indicators related to sarcosine, cholesterol esters (CE 20:0, 20:1, 22:2) and increased arachidonic acid, acylcarnitine (C12) in the BCG-positive diabetic group. **Figures 3** shows examples of significantly different levels of these metabolites. Enrichment analysis revealed changes in cholesterol esters pathway in BCG positive patients compared to BCG negative patients at the nominal level of significance (p < 0.01).

**Table 2:** Metabolites associated with BCG vaccination in diabetic patients with COVID-19.

Names	pathway	Estimate	Standard Error	p-value
Sarcosine Synthesis from Glycine (Sarcosine/Glycine)	Metabolism Indicators	-0.856	0.259	0.003
Cer (d18:1/26:1)	Ceramides	0.556	0.186	0.006
Sarcosine Synthesis from Choline (Sarcosine/Choline)	Metabolism Indicators	-0.634	0.216	0.006
Sarcosine	Amino acids Related	-0.587	0.208	0.009
C12	Acylcarnitine	0.297	0.105	0.010
CE (20:0)	Cholesterol Esters	-1.049	0.379	0.012
CE (22:2)	Cholesterol Esters	-0.862	0.340	0.021
Valinemia (NBS)	Metabolism Indicators	-0.347	0.143	0.022
CE (20:1)	Cholesterol Esters	-0.650	0.278	0.027
TG (16:0_28:2)	Triacylglycerols	-0.914	0.408	0.033
Hex3Cer(d18:1_20:0)	Glycosylceramides	0.339	0.152	0.033
Ratio of AC-OHs to ACs (Ratio of Hydroxylated Acylcarnitines to Acylcarnitines)	Metabolism Indicators	1.174	0.511	0.034
AA (Arachidonic acid)	Fatty Acids	0.409	0.184	0.034
MCKAT Deficiency (NBS)	Metabolism Indicators	-0.201	0.076	0.046
DLD (NBS)	Metabolism Indicators	-0.409	0.202	0.05
Aconitic Acid	Carboxylic Acids	-0.418	0.204	0.05

### 3.4 Metabolites associated with BCG vaccination in non-T2D patients

**Figure 4:**
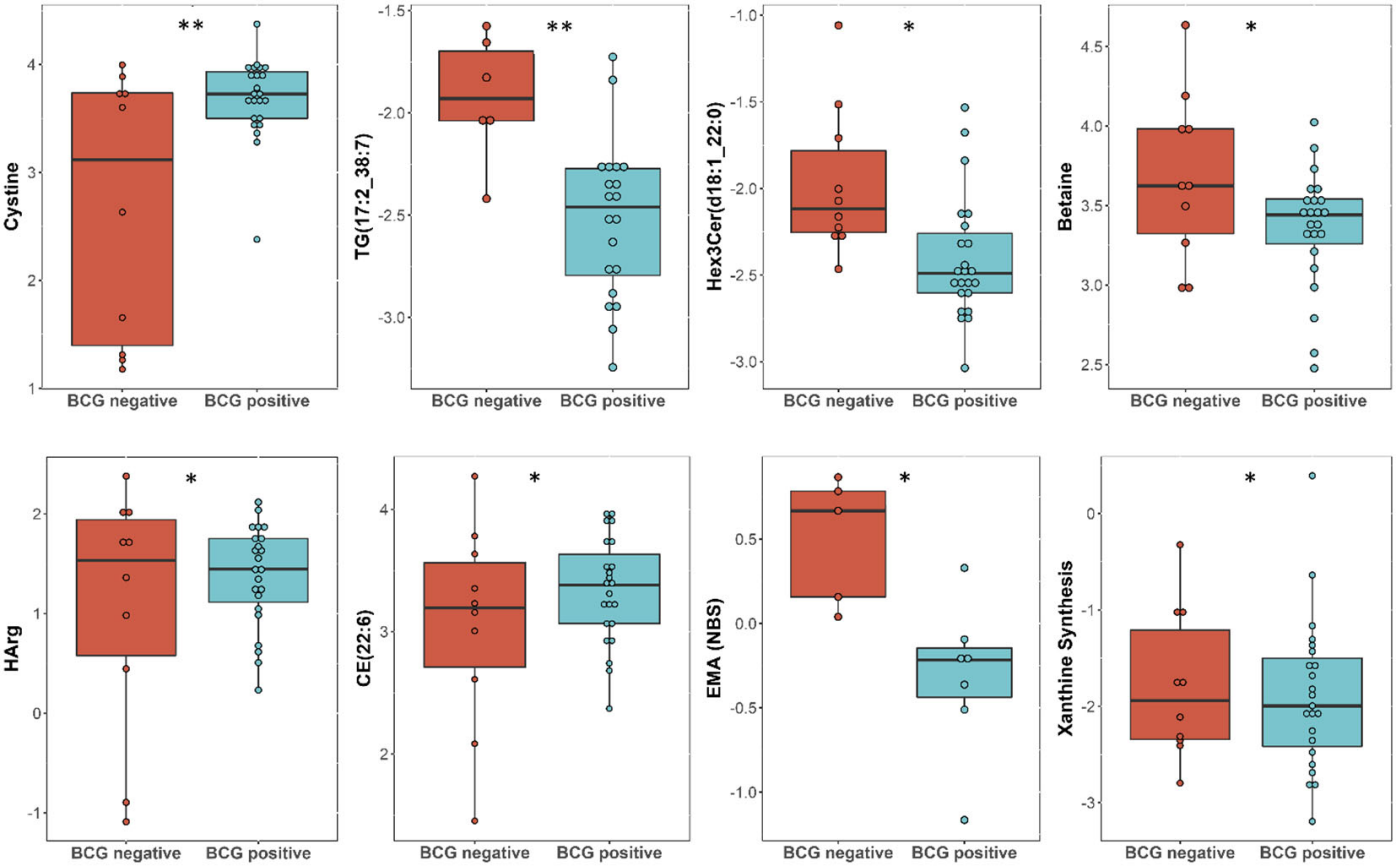
Metabolites associated with BCG vaccination in non-T2D patients with different levels of COVID-19 severity. **/* signifies <0.01/<0.05 p value.

Linear regression revealed several nominally (≤0.001) significant changes between the vaccinated and non-vaccinated groups (**Table 3**). These alterations include the difference in the levels of various glycosylceramides, triacylglycerols, amino acid related metabolites such as cystine, HCys, alpha-aminobutyric acid (AABA), and Betaine. **Figure 4** shows examples of the top nominally significant levels of these based on vaccination status in non-diabetic patients. Enrichment analysis revealed changes in the glycosylceramide pathway in BCG vaccinated individuals compared to non-vaccinated counterpart (p = 0.01). Other significant pathways include amino acid related metabolites (p = 0.015).

**Table 3:** Metabolites associated with BCG vaccination in non-diabetic patients with COVID-19.

Name	pathway	Estimate	Standard Error	P value
Cystine	Amino acids Related	1.178	0.289	3.08E-04
Cystine Synthesis	Metabolism Indicators	0.944	0.249	0.001
TG (17:2_38:7)	Triacylglycerols	-0.677	0.192	0.002
LysoPC a C26:0	Glycerophospholipids	0.305	0.002	0.004
HCys Synthesis	Metabolism Indicators	-0.493	0.160	0.005
TG (22:3_30:2)	Triacylglycerols	-0.409	0.121	0.005
Ratio of SFAs to FAs	Metabolism Indicators	-0.563	0.166	0.007
3-IAA	Indoles Derivatives	-0.647	0.237	0.011
CE (22:0)	Cholesterol Esters	-0.920	0.107	0.013
BABA Synthesis	Metabolism Indicators	-1.893	0.402	0.018
Betaine	Amino acids Related	-0.418	0.169	0.019
Ratio of MUFAs to FAs	Metabolism Indicators	-0.393	0.146	0.020
EMA (NBS)	Metabolism Indicators	-0.754	0.270	0.021
Spermidine Synthesis	Metabolism Indicators	0.587	0.199	0.022
Hex3Cer(d18:1_22:0)	Glycosylceramides	-0.372	0.154	0.022
Hex2Cer(d18:1/22:0)	Glycosylceramides	-0.236	0.098	0.022
CE (15:1)	Cholesterol Esters	-0.406	0.155	0.024
AABA (α-aminobutyric acid)	Amino acids Related	0.473	0.204	0.027
Sum of Aminobutyric Acids	Metabolism Indicators	0.433	0.192	0.031
HCys (Homocysteine)	Amino acids Related	-0.320	0.144	0.035
TG (17:2_38:5)	Triacylglycerols	-0.477	0.214	0.035
HexCer (d18:1/26:1)	Glycosylceramides	-0.269	0.122	0.036
DG (18:2_20:0)	Diacylglycerols	-0.288	0.125	0.040
Hex2Cer (d18:1/24:0)	Glycosylceramides	-0.211	0.100	0.042
MTHFR Deficiency (NBS)	Metabolism Indicators	0.218	0.103	0.043
PC aa C42:4	Glycerophospholipids	-0.218	0.103	0.043
Spermidine	Biogenic Amines	0.570	0.237	0.047
HexCer (d18:1/22:0)	Glycosylceramides	-0.299	0.146	0.049
GUDCA	Bile Acids	-0.885	0.432	0.05
3-IAA Synthesis	Metabolism Indicators	-0.588	0.286	0.05
LysoPC a C26:1	Glycerophospholipids	0.164	0.081	0.05

### 3.5 Spearman’s correlation between clinical traits and significant metabolites in diabetic COVID-19 patients.

**Figure 5:**
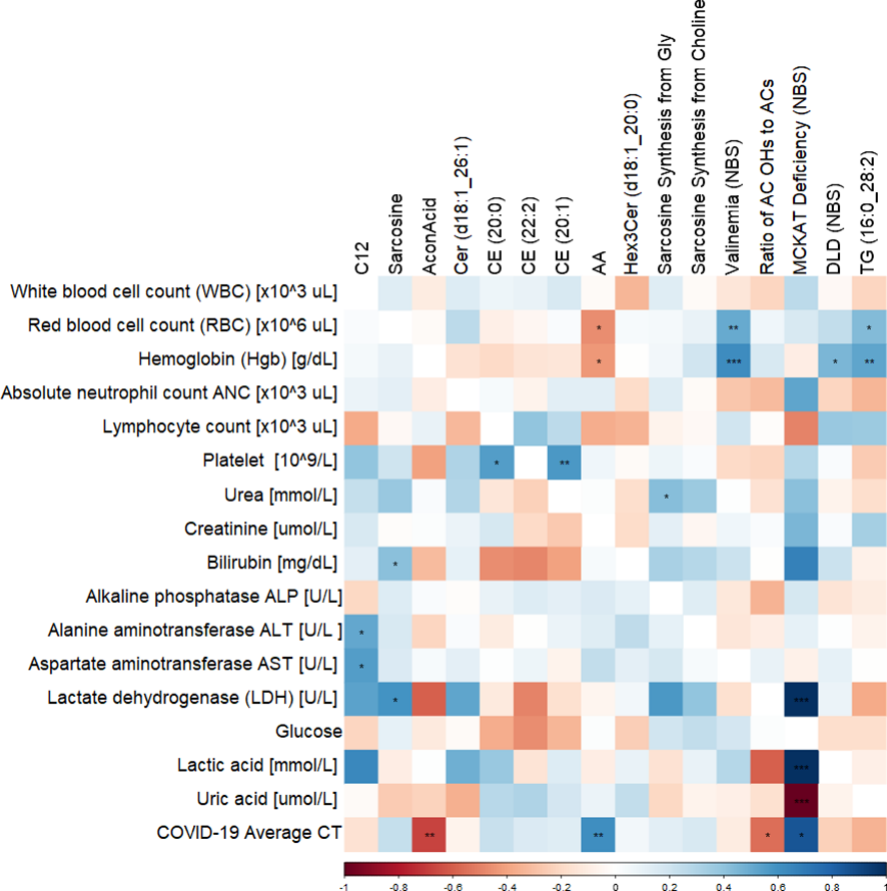
Correlation matrix showing the Spearman’s association between the significant metabolites and clinical traits in BCG-positive diabetic individuals. ***/**/* signifies <0.001/<0.01/<0.05 p value.

Correlation of clinical measurements of diabetic BCG-positive individuals and significantly altered metabolites using Spearman’s test revealed negative association of aconitic acid (p<0.01) and positive correlation of arachidonic acid (p<0.01) with SARS-CoV-2 infection average RT-PCR cycle threshold (CT). Arachidonic acid also shows negative association with hemoglobin and red blood cells (RBC) levels (p<0.05). The amino acid sarcosine shows positive correlation with bilirubin (p<0.05), lactate dehydrogenase (p<0.05), and average CT (p=ns). Data are summarized in **Figure 5**.

## 4 Discussion

When a virus infects a human body, such as SARS-CoV-2, both the host and the virus influence each other’s metabolism. In fact, host cells support viral survival and reproduction, and as a result, undergo metabolic reprogramming (26). This metabolic alterations can affect the host’s immune response, resulting in a wide spectrum of outcomes, from asymptomatic illness to life-threatening respiratory distress syndrome, and death (25). Additionally, severity of the illness and mortality was closely associated with pre-existing comorbidities such as cardiovascular disease, hypertension, diabetes etc. (27, 28). During the early stages of vaccine development for COVID-19, it was reported that countries with neonatal BCG vaccination policy had fewer cases and lower mortality rate than the countries without BCG (29, 30), sparking interest in the cross-protective nature of BCG vaccine. Since then, several studies have only hypothesized on the safety of BCG for COVID-19 (2, 14, 15, 31).

BCG vaccine is suggested as a prophylactic adjuvant to decrease the severity of COVID-19 when no other agents are available (14). In order to understand if the BCG vaccine can help in reducing the sickness in high-risk group such as the T2D individuals, this study aimed to compare the effect of BCG vaccination status and investigate the metabolic signature associated with BCG vaccination in diabetic vs non-diabetic COVID-19 patients.

In this retrospective pilot study of 67 COVID-19 patients, BCG vaccinated T2D patients (25, 96%) presented with higher severity than BCG-negative T2D (5, 62.5%) patients. This study aimed to elucidate the potential metabolic differences between BCG positive T2D patients with COVID-19 and its BCG-negative counterpart, which could provide an insight into the underlying molecular mechanisms responsible for increased risk of disease progression. The discovery of these pathways would offer more information about the efficacy of BCG vaccination for COVID-19 disease management in T2D individuals.

In this study, targeted metabolomics of serum samples from T2D COVID-19 patients with different levels of disease severity has revealed alterations in amino acid sarcosine, cholesterol esters, and ceramides in BCG-positive group. Functional enrichment analysis revealed changes in cholesterol esters is associated with BCG vaccination in diabetic individuals with COVID-19 (**Figure 2C**, **Table 3**). Lower level of sarcosine, metabolic indicators (sarcosine/glycine and sarcosine/choline ratios) was observed in the BCG positive diabetic group compared to BCG-negative counterpart. Sarcosine is an amino acid that is crucial in COVID-19 pathology as it is responsible for boosting antigen-presenting cell activity (32) and autophagy (33), the body’s process of eliminating damaged cells and their immunostimulatory waste. In the course of COVID-19, autophagy functions as a protective catabolic process and is essential for the antiviral response *via* the direct removal of virus, the display of viral antigens, and the suppression of excessive inflammation (34). Furthermore, our data revealed that arachidonic acid (AA) was elevated in BCG-positive diabetic population. AA is highly potent antiviral agent that renders enveloped viruses, such as SARS-CoV-2 inactive by suppressing viral replication (35). Previous studies have also shown that AA pathway up-regulated due to BCG vaccination (36). Other COVID-19 specific ceramides-related metabolite Cer(d18:1/26:1) (37) was found to be increased in BCG positive in this study.

The non-T2D (general population) counterpart showed no statistical significant difference between BCG vaccinated (60%) and non-BCG vaccinated (70%) groups with COVID-19 severity, which was consistent with clinical trial in this study (31). However, when closely looked at the metabolic profile difference between the study groups, alterations in glycosylceramides, amino acid related metabolites and triacylglycerols were observed in the BCG vaccinated group. Functional enrichment analyses showed glycosylceramides enriched in the BCG vaccinated group (**Figure 2D**, **Table 3**). Glycosylceramides (Hex3Cer(d18:1_22:0), Hex2Cer(d18:1/22:0), HexCer(d18:1/26:1), Hex2Cer(d18:1/24:0), HexCer(d18:1/22:0)) were lower in the BCG vaccinated non-diabetic population, as previously suggested to be lower in severe COVID-19 patients (37). Higher levels of glycerophospholipids (Lyso PC a 26:0, lyso PC a 26:1) and triacylglycerols shown here are also consistent with previous studies that showed critically ill patients having increased levels of these metabolites (37, 38). The results in this study are on par with the published metabolic profile of severe COVID-19 patients. However, interestingly, increased level of spermidine and metabolic indicator spermidine synthesis from polyamine putrescine are seen in BCG vaccinated individuals. Spermidine has cardio-protective (39), anti-inflammatory and antioxidant properties (40, 41), which may be mediated *via* autophagy (42, 43). Indeed, studies have suggested the therapeutic effects of spermidine in COVID-19 affected individuals (44, 45). Additionally, viral replication blocks the conversion of putrescine to spermidine, preventing autophagy and slowing the hosts’ immune response (45). In this study, the increase of spermidine and spermidine conversion from putrescine suggests a conceivably positive effect of BCG in non-diabetic individuals as polyamine metabolism is altered by BCG vaccination (36), possibly due to innate immunity training mechanism.

Spearman’s correlation of clinical parameters and metabolites altered in BCG vaccinated group among diabetic individuals shows negative correlation between AA and SARS-CoV-2 average CT, hemoglobin and RBC measurements. As previously mentioned, higher value of AA is associated with suppressing viral infection, perhaps explaining the positive association between AA and average CT and lower viral load, although the negative association with hemoglobin and RBC levels could indicate increased oxidative stress exerted by high levels of AA in the BCG vaccinated patients (46). Sarcosine shows a positive association with average CT, although not significant, which indicates lower viral load with higher level of sarcosine. It also has a positive correlation with lactate dehydrogenase (LDH), a biomarker of COVID-19 severity and mortality (47). The latter association requires further investigation.

**Figure 6:**
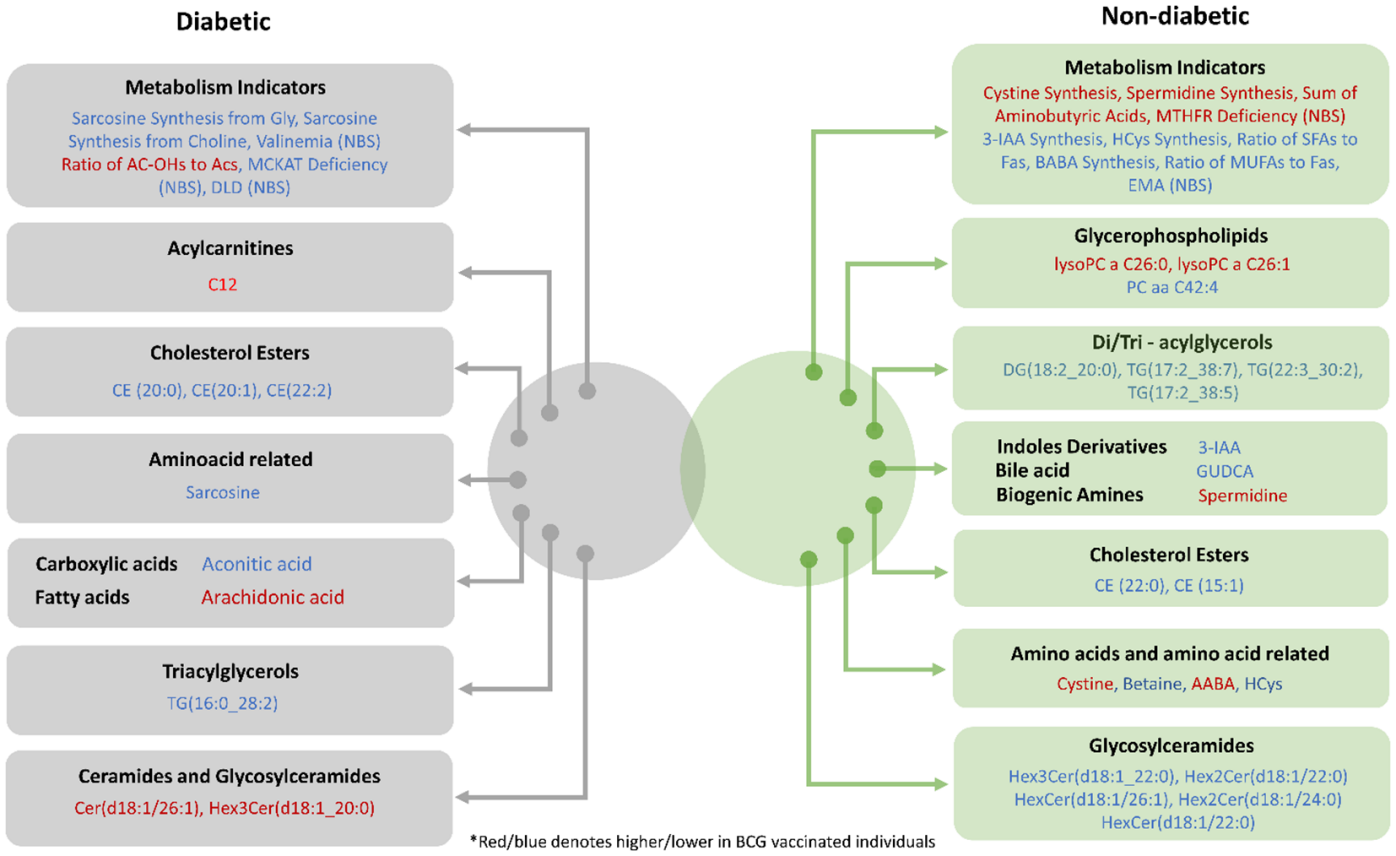
Venn diagram providing a snapshot of the BCG associated metabolites from diabetic and non-diabetic individuals with varying levels of COVID-19 severity.

In both diabetic and non-diabetic patients, most of the metabolites identified in the BCG vaccinated cohort were also identified with COVID-19 patients in general population. Sarcosine, AA, and spermidine metabolites and their altered levels in the experimental groups suggest a difference in the efficacy of BCG vaccination for the management of diabetic COVID-19 patients (**Figure 6**). It must still be determined whether these alterations are mediators or consequences of the BCG vaccination.

## 5 Study limitations

There are several drawbacks in this study including the low number of participants in control groups (BCG negative) and the retrospective nature of the study, which has restricted the information required for interpreting the data from a pathological perspective. Additionally, the study cohort were predominantly males (62 males and 5 females), further studies are required with gender matched subjects to conclude that the findings reflect the metabolic profile of the whole general population. It is possible that other factors and comorbidities with T2DM, conditions such as tobacco usage, COPD, and pneumonia may have contributed to the results seen in the study population. Additionally, the duration between the BCG re-vaccination (if applicable) and SARS-CoV-2 infection was not corrected for in the analysis. Further research is required on a large-scale population which includes information on the patient medication history, comorbidities, smoking habits, and respiratory illness in order to determine the mechanistic involvement of the specific compounds/metabolites mentioned in this study. Nevertheless, to the best of our knowledge, no other study has highlighted the likelihood of negative effect of BCG in T2D individuals with COVID-19.

## 6 Conclusions

To conclude, our data indicates a possible change in the metabolic profile of diabetic patients with previous BCG vaccination compared to non-vaccinated patients. Our data highlights a possibility that BCG vaccination may have detrimental effect on the diabetic population with regard to COVID-19 severity.

## Data availability statement

The raw data supporting the conclusions of this article will be made available by the authors, without undue reservation.

## Ethics statement

The studies involving human participants were reviewed and approved by Institutional Review Boards (IRBs) of HMC (MRC-01-20-145) and Qatar University (QU-IRB 1289-EA/20). The patients/participants provided their written informed consent to participate in this study.

## Author contributions

NA, MEl designed and performed the data analysis. NA wrote the manuscript. MEl supervised and designed the study and edited the manuscript. FC, HY, AA-T, AA, and MEm helped in the sampling and collection of patients information. All authors contributed to the article and approved the submitted version.
